# The ins and outs of HIV restriction

**DOI:** 10.1186/1742-4690-10-S1-O30

**Published:** 2013-09-19

**Authors:** Áine McKnight

**Affiliations:** 1 Centre for Immunology and Infectious Disease, Blizard Institute, Barts and The London School of Medicine and Dentistry, Queen Mary University of London, 4 Newark Street, London E1 2AT, UK

## Background

The interaction of viruses with their human host is a constant war. After the virus infiltrates the body the journey to target cell is fraught with danger. Once it interacts with the target cell a new battle, against the cell’s intrinsic factors that restrict replication, begins. For human immunodeficiency virus (HIV) several restriction factors, including APOBEC3 (apolipoprotein-B-mRNA-editing-enzyme), SAMHD1, p21 and tetherin are described.

## Materials and methods

An siRNA screen of ~20,000 human genes was used to uncover those involved in inhibition of HIV replication.

## Results

114 genes were identified to be potentially involved in intrinsic resistance. The mechanism of restriction for several of these genes (AP2M1, dynamin and SETDB1) had been previously described. AP2M1 and dynamin are involved in an entry dependent restriction Lv2, while SETDB1 is part of a pre-integration restriction also involving TRIM28. Many factors were known to be involved in receptor signaling, vesicle trafficking, transcription, apoptosis, cross-nuclear membrane transport, meiosis, DNA damage repair, ubiquitination and RNA processing. Focusing on the most potent factors led us to discover an anti-viral role for the PAF1 complex (RNA polymerase II associated factor, PAF1c). PAF1c has been previously implicated in gene transcription, cell cycle control and mRNA surveillance [1] and was later shown to be also antiviral for influenza virus [2]. Expression of PAF1 in host cells renders them refractory to HIV-1, -2 and Simian Immunodeficiency Virus infection. PAF1c is expressed in monocytes, macrophages and CD4+ T-lymphocytes and we demonstrated potent activity in MonoMac1, a monocyte cell line.

RNA-associated Early-stage Anti-viral Factor (REAF), annotated in the human genome but with no known function, was also identified to restrict HIV replication at an early stage during reverse transcription. REAF interacts (either directly or indirectly) with HIV RNA or RNA:DNA intermediates during reverse transcription. Also, during the process of reverse transcription REAF protein is degraded, within one hour of infection, in a proteosomal dependent manner.
